# Relationship between Chinese medicine dietary patterns and the incidence of breast cancer in Chinese women in Hong Kong: a retrospective cross-sectional survey

**DOI:** 10.1186/s13020-017-0138-9

**Published:** 2017-06-29

**Authors:** Xiao Zheng, Jianping Chen, Ting Xie, Zhiyu Xia, Wings Tjing Yung Loo, Lixing Lao, JieShu You, Jie Yang, Kamchuen Tsui, Feizhi Mo, Fei Gao

**Affiliations:** 1 School of Chinese Medicine, The University of Hong Kong, Hong Kong, China; 2 Department of Dermatology, Guangzhou University of Chinese Medicine, Guangzhou, 510020 China; 3 School of Public Health, Peking University, Beijing, China; 4 The Hong Kong Associate of Chinese Medicine, Hong Kong, China; 5 Chengdu University of Traditional Chinese Medicine, Chengdu, 510020 China

## Abstract

**Background:**

This retrospective cross-sectional study aimed to investigate the relationship between Chinese medicine (CM) dietary patterns (*hot*, *neutral*, and *cold*) and the incidence of breast cancer among Chinese women in Hong Kong.

**Methods:**

Breast cancer cases (n = 202) and healthy controls (n = 202) were matched according to demographics. Chinese women residing in Hong Kong for the past 7 years were recruited by media advertisements (e.g., via newspapers, radio, and posters). The control participants were recruited by convenience sampling from health workshops held in clinics and communities of 15 districts of Hong Kong. After completing test–retest reliability, all participants were asked to complete diet pattern questionnaires about their food preferences and dietary patterns. The Student’s unpaired *t* test, Chi square test, and logistic regression were conducted using SPSS software.

**Results:**

Three major CM dietary patterns were identified: *hot*, *neutral*, and *cold*. The participants with breast cancer exhibited a stronger preference for *hot* food than the control group (Chi square test, *P* < 0.001). A higher frequency of breast cancer was associated with a higher frequency of dining out for breakfast (4–5 times per week, Chi square test, *P* = 0.015; 6–7 times per week, Chi square test, *P* < 0.001) and lunch (4–5 times per week, Chi square test, *P* < 0.001; 6–7 times per week, Chi square test, *P* = 0.006). The participants with no history of breast cancer consumed CM supplements and Guangdong soups (1–2 times per week, Chi square test, *P* = 0.05; >3 times per week, Chi square test, *P* < 0.001) more frequently than those with breast cancer.

**Conclusions:**

Non-breast cancer participants adopted a *neutral* (healthy and balanced) dietary pattern, and consumed CM supplements and Guangdong soups more frequently.

**Electronic supplementary material:**

The online version of this article (doi:10.1186/s13020-017-0138-9) contains supplementary material, which is available to authorized users.

## Background

Breast cancer is the most common and one of the most fatal cancers in women worldwide. The number of breast cancer cases among women in Hong Kong increased from 2273 in 2004 to more than 2870 in 2009, and average incidence rate per 100,000 women between 2004 and 2009 was 386.7 [1]. In 2008, the lifetime risk of breast cancer was 1 in 20 women [2]. Breast cancer incidence was generally lower in Asia than in Europe in 2000 [3]; a key reason for this was the difference in dietary patterns between the East and the West [4]. The identification of reasonable dietary prevention methods and the development of corresponding inhibitors may be beneficial for breast cancer prevention [5]. However, little research has been conducted in this area.

The dietary patterns of women in Hong Kong have been changing with western lifestyle in recent years [6, 7]. The intake of foods with certain properties (e.g., *hot* or *cold* features) may increase the risk of related diseases, such as cancers [8]. Although diet has been proposed as a possible cause of breast cancer [9–11], the incidence of breast cancer has not previously been related to Chinese medicine (CM)-defined dietary patterns.

The properties of CM material medica (*cold*, *cool*, *hot*, and *warm*) can be used to classify diets as *hot*, *neutral*, or *cold*; these types of food have different functions in the body. Under this classification, beef, shrimp, chicken, and even ice cream are *hot* foods (Additional file 1).

This study aimed to investigate the relationship between CM dietary patterns and the incidence of breast cancer among women in Hong Kong.

## Methods

### Study design and participants

A retrospective cross-sectional survey was conducted. Data were collected using the Food Frequency Questionnaires (FFQ) (Additional file 2) [12]. The selection of study participants was based on geographic and sociodemographic variables described previously [6, 8, 9]. The Minimum Standards of Reporting Checklist (Additional file 3) contain details of the experimental design, and statistics, and resources used in this study.

This study included female participants with breast cancer (cases), who were compared with healthy women (controls). The cases were recruited by advertisements in the local media, such as in newspapers, in booklets issued by the University of Hong Kong, on posters, and on the radio. Convenience sampling was used to recruit the control group from health workshops in clinics and communities in 15 Hong Kong districts. All participants had to fulfill the following conditions: (1) female, (2) Chinese women residing in Hong Kong, (3) 28–60 years of age, (4) living in Hong Kong for the past 7 years, (5) able to understand the questionnaire items in Chinese, and (6) provision of an informed consent. Controls were required to fulfill the following conditions: (1) no breast cancer or other gynecologic diseases; (2) no metabolic, nutritional, or other severe diseases; and (3) no serious disease after doctor consultation in the past 3 months. Case inclusion criteria were: (1) new primary breast cancer patients, and (2) a diagnosis of primary breast cancer through biopsy and/or discharge report. Participants were excluded from the study if they had (1) other prior cancer diagnosis, (2) treatments with psychotropic drugs, (3) any medical conditions that limited their physical activity, and (4) not completed the questionnaire.

### Sample size

We conducted a pilot study with 37 participants whose total diet comprised mainly *hot* foods and an expected odds ratio (OR) of 2.32 according to the formula of OR was found. For the pilot study, we invited 37 people from the oncology outpatient clinic and the general outpatient clinic in the School of Chinese Medicine, University of Hong Kong, to complete the questionnaire: 20 were breast cancer cases and 17 were not. After the pilot study, we analyzed the responses and calculated the consumption of 12 kinds of *hot* foods including beef, mutton, shrimp, soft drinks, and milk. Of the breast cancer cases, 45.8% consumed *hot* foods regularly, whereas only 26.7% of the controls did so. We chose 10% as the estimated prevalence of *hot* food consumption for control group and calculated the estimated prevalence of *hot* food consumption for breast cancer group as 0.2 the expected OR of 2.32 to estimate the sample size of our research. The estimated sample size was calculated as about 207 participants in each group. Taking into account the nullified questionnaires, 202 participants were eventually included out of the total 207 participants recruited in each group.

### Questionnaire

A self-administered FFQ was used to collect the dietary intake information. This questionnaire was adapted from the Fred Hutchinson Cancer Research Center Food Questionnaire, which is based on Western dietary patterns [13, 14]. Two CM experts and a group of laypeople approved the revised questionnaire as suitable to assess the average dietary patterns of Chinese women in Hong Kong. The FFQ consisted of 74 food items (Additional file 4). A commonly used portion size was specified for each food based on the conventional food intake and dietary patterns of Hong Kong women.

The questionnaire consists of two sections. The first section assessed sociodemographic information, including age, working status, education level, weight, height, and marital status. The second section contained the CM FFQ items, which measured consumption of very common foods. The FFQ consists of 74 items scored on a 5-point scale, ranging from 1 (not consumed at all) to 5 (consumed very often). It comprises nine subscales of food consumption frequency for CM-defined foods, and a general preference score for three kinds of food (*cold*, *neutral*, and *hot* foods, classified according to CM). A total score for each subscale was obtained by summing the relevant item scores. Higher scores indicated higher consumption frequencies [15]. We modified the semantic expression of several items to adapt those items to the dietary patterns and habits of the women in Hong Kong. Our pilot study of 37 participants demonstrated that the version could be read and understood by local Chinese women.

The questionnaire design was single-blind to participants to ensure the validity of participants’ stated consumption of their preferred and most frequently eaten foods. Different kinds of food were included and randomized to ensure the aim of the survey was not revealed to the cases or controls. Completed questionnaires were collected individually to ensure the responses were not misinterpreted owing to communication gaps between the researchers and the participants.

### Study procedures

Test–retest reliability was examined using pre-survey responses. Two weeks after completing the first questionnaire, we asked the same participants to complete the same questionnaire to confirm the instrument’s validity and test–retest reliability. Analysis of homogeneity in this pilot study result was used to test the consistency of the 37 completed questionnaires (20 cases and 17 controls). The results produced a *P* value of 0.915, which indicated that the questionnaire responses were statistically the same for the two tests. Each participant was asked to appraise each item for its relevance in evaluating the content, and to suggest any items that should be added to improve the questionnaire’s validity. All items had a content validity index of clarity greater than 80%.

Every potential participant was interviewed by a trained research doctor at the clinic center of the School of Chinese Medicine, University of Hong Kong. Participants completed a self-administered questionnaire and had a face-to-face consultation for the same research doctor to check the questionnaire.

Three important steps were taken throughout the process to ensure the quality of investigation. First, to ensure consistency of the survey across sites and over time, the same interviewer used the same procedures and standards to investigate all women. Second, each questionnaire was checked by the same researcher carefully and those questionnaires with missing data on foods or dietary patterns were excluded. Third, to prevent input error, we used a double data entry method by different researchers to enter all data into SPSS.

### Measurement

According to CM theory, different foods possess different *cold*, *neutral*, and *hot* properties and have different nutritional constituents, each with their own *cold*, *neutral*, and *hot* properties. These food properties are based on the functions the foods perform after consumption. All vegetables, meat, fruits, and grains are classified according to one of these three properties, as summarized in Additional file 1. Participants’ final overall food preference score of *hot*, *neutral*, or *cold* was determined by the average score for all the foods listed. For example, if the overall FFQ score for *neutral* food was the highest, then the overall preference score for food was considered *neutral*. The *hot* and *cold* preference scores were calculated in the same manner.

Additionally, participants were asked to indicate the frequency of (1) eating out for breakfast, lunch, and dinner (0–1 time/week; 2–3 times/week; 4–5 times/week, and 6–7 times/week); (2) CM intake, CM supplement intake, and Chinese soup consumption (seldom, sometimes, often); and (3) Chinese-style food (i.e., Guangdong *dim sum*/tea drinking) and Western-style food consumption (i.e., fast food) (<once/week; 1–2 times/week; >3 times/week). Demographic information such as age, marital status, current height and weight (to obtain body mass index), and education level was also collected.

### Data analysis

The statistics program SPSS version 16.0 (SPSS Inc., Chicago, IL, USA) was used for data analysis. Descriptive statistics were calculated; continuous variables were presented as mean ± standard deviation, and categorical variables were presented as frequencies. The mean difference between continuous variables was tested using Student’s *t* test for independent samples. The association between categorical variables was tested using the Chi square test. A binary logistic regression model was used to evaluate the association of different food preferences with the likelihood of having breast cancer. Results were presented as ORs and corresponding 95% confidence intervals, with age as a variable in the logistic regression. All *P* values were based on two-tailed tests, with *P* < 0.05 considered statistically significant.

Regarding the validity and reliability assessment of the questionnaire, we examined test–retest reliability by inviting participants in the pilot study to complete the questionnaire a second time. We compared both test results using ANOVA, which produced a value of 0.915, showing high test–retest reliability. All the pilot study participants were interviewed about the interpretability of the questionnaire, and the interpretability was reported to be good.

## Results

### Sociodemographic characteristics of participants

The case and control groups were similar in terms of body mass index, occupation, education, marital status, age at first menstrual cycle, and family history of cancer (Table 1).

**Table 1:** Socio-demographic characteristics of participants (*N* = 404,%)

Socio-demographic characteristics	Controls (*n* = 202)	Cases (*n* = 202)	*P* values
Age			0.061
Mean ± SD	45.35 ± 6.765	47.75 ± 6.17	
≤34	14 (6.9)	7 (3.5)	
35–41	39 (19.3)	25 (12.4)	
42–48	76 (37.6)	72 (36.6)	
49–55	62 (30.7)	78 (39.0)	
≥56	11 (5.4)	18 (9.0)	
BMI			0.132
Underweight (<18.5)	17 (8.5)	19 (9.5)	
Normal weight (18.5–24.9)	143 (71.1)	156 (78.4)	
Overweight (25–29.9)	33 (16.4)	21 (10.6)	
Obesity (≥30)	8 (4)	3 (1.5)	
Occupational type			0.589
Full-time	119 (59.5)	113 (55.9)	
Part-time	14 (7.0)	22 (10.9)	
Housewife	66 (33.0)	66 (32.7)	
Others	1 (0.5)	1 (0.5)	
Educational level			0.063
Primary	21 (10.4)	24 (11.9)	
Secondary	92 (45.5)	115 (56.9)	
Tertiary or above	87 (43.1)	62 (30.7)	
Others	2 (1.0)	1 (0.5)	
Marital status			0.389
Single	33 (16.3)	25 (12.4)	
Married or cohabited	155 (76.7)	158 (78.2)	
Divorced	14 (6.9)	19 (9.4)	
Family tumor history			0.318
Yes	87 (43.1)	97 (48.0)	
No	115 (56.9)	105 (52.0)	

*BMI* body mass index. The following factors are related to breast cancer incidence and were measured by the questionnaire: age, race, BMI, educational level, marital status, religion, family tumor history, and occupation. The two groups were comparable on these factors, although no significant between-group differences were found

When age was treated as a continuous variable, there was a significant difference between the case and control groups (*P* < 0.001): participants in the control group were 2.4 years younger. However, when age was categorized according to five different age groups, there was no significant difference between the case and control groups (*P* = 0.061). Because there was a marginally significant difference in age between the two groups, we considered age a confounder in multivariate analysis of variance (MANOVA).

### Characteristics of food preferences based on CM theory

Tables 2 and 3 showed that there was a significant between-group difference in the frequency distribution of the preferences for the three food types (*P* < 0.001). We analyzed the between-group differences in food preferences of *hot*, *neutral*, and *cold* using logistic regression with age as a variable. The consumption of *hot* foods such as roast meat, shrimp, shellfish, soft drinks, popcorn/fries, and candies was significantly different between groups. Participants with a strong preference for roast meat, shrimp, shellfish, and popcorn/fries had 6-, 9.2-, 13.3-, and 7.6-times higher risk, respectively, of having breast cancer compared with participants with an average preference for these foods. Women who preferred soft drinks had a 4.9-times higher risk of having breast cancer compared with women who had an average preference. Higher preferences for nuts and freshwater fish (*neutral)* and tofu and squid (*cold)* showed a lower OR for breast cancer (Table 4).

**Table 2:** Frequency distribution of preference for foods with hot, neutral and cold characteristics (N = 404, %)

Groups	Food nature			*P* value
	Hot (warm)	Neutral	Cold (cool)	
Controls	14 (6.9)	121 (59.9)	67 (33.2)	<0.001*
Cases	53 (26.4)	103 (50.8)	46 (22.8)	

The *P* value was <0.05, indicating a statistically significant between-group difference. The results showed a possible association between consumption of *hot* foods and a higher risk of breast cancer

* *P* value was calculated by Chi square test

**Table 3:** Specific frequency distribution of preference for foods with *hot*, *neutral* and *cold* characteristics (*N* = 404, %)

Food nature	Food	Breast cancer	Very preferred	Preferred	Average	Not preferred	Not very preferred	Statistical significance
*Hot*	Spicy food	No	14 (6.9)	50 (24.8)	82 (40.6)	43 (21.3)	13 (6.4)	0.655
		Yes	19 (9.4)	55 (27.2)	68 (33.7)	46 (22.8)	14 (6.9)	
	Roast meat	No	5 (2.5)	59 (29.2)	113 (55.9)	22 (10.9)	3 (1.5)	0.237
		Yes	12 (5.9)	68 (33.7)	104 (51.5)	17 (8.4)	1 (0.5)	
	Beef	No	16 (8.0)	58 (28.9)	83 (41.3)	29 (14.4)	15 (7.5)	0.534
		Yes	24 (12.1)	48 (23.6)	87 (43.2)	31 (15.1)	12 (6.0)	
	Mutton	No	8 (4.0)	32 (16.0)	83 (41.0)	50 (24.5)	29 (14.5)	0.777
		Yes	11 (5.6)	33 (16.3)	72 (35.7)	51 (25.0)	35 (17.3)	
	Chicken	No	36 (17.8)	102 (50.5)	55 (27.2)	8 (4.0)	1 (0.5)	0.138
		Yes	56 (27.8)	82 (40.4)	54 (26.8)	8 (4.0)	2 (1.0)	
	Salmon/tuna	No	30 (14.9)	87 (43.3)	57 (28.4)	19 (9.5)	7 (3.5)	0.687
		Yes	37 (18.2)	76 (37.4)	65 (32.3)	18 (9.1)	6 (3.0)	
	Shrimp	No	13 (6.5)	53 (26.4)	84 (41.3)	38 (18.9)	14 (7.0)	*<0.001* *
		Yes	44 (21.9)	60 (29.9)	73 (6.3)	19 (9.5)	5 (2.5)	
	Shellfish	No	9 (4.5)	37 (18.3)	97 (48.0)	48 (23.8)	11 (5.4)	*<0.001* *
		Yes	31 (15.2)	56 (27.8)	73 (36.4)	39 (19.2)	3 (1.5)	
	Dark Chinese tea	No	30 (14.9)	76 (37.6)	73 (36.1)	15 (7.4)	8 (4.0)	0.071
		Yes	31 (15.2)	73 (36.0)	67 (33.0)	30 (14.7)	2 (1.0)	
	Coffee/tea with milk	No	38 (18.8)	52 (25.7)	52 (25.7)	44 (21.8)	16 (7.9)	0.678
		Yes	43 (21.5)	54 (26.5)	56 (27.5)	32 (16.0)	17 (8.5)	
	Soft drinks	No	3 (1.5)	10 (5.0)	65 (32.2)	92 (45.5)	31 (15.3)	*0.001* *
		Yes	10 (5.0)	30 (14.9)	72 (35.8)	65 (32.3)	24 (11.9)	
	Milk	No	10 (5.0)	49 (24.4)	91 (44.8)	35 (17.4)	17 (8.5)	*0.014* *
		Yes	25 (12.2)	37 (18.3)	90 (44.7)	43 (21.3)	7 (3.6)	
	Alcohol	No	0 (0.0)	4 (2.0)	21 (10.4)	58 (28.7)	117 (57.9)	0.257
		Yes	3 (1.5)	2 (1.0)	17 (8.5)	66 (32.5)	114 (56.5)	
	Beer	No	2 (1.0)	23 (11.4)	64 (31.7)	48 (23.8)	64 (31.7)	0.075
		Yes	6 (3.0)	15 (7.5)	47 (23.1)	65 (32.2)	69 (34.2)	
	Popcorn/fries	No	3 (1.5)	34 (16.8)	79 (39.1)	57 (28.2)	29 (14.4)	*0.035* *
		Yes	15 (7.6)	32 (15.7)	86 (42.4)	46 (22.7)	23 (11.6)	
	Chocolate	No	25 (12.4)	61 (30.3)	82 (40.8)	26 (12.9)	7 (3.5)	0.842
		Yes	25 (12.4)	64 (31.8)	72 (35.8)	31 (15.4)	9 (4.5)	
	Candies	No	3 (1.5)	21 (10.4)	93 (46.0)	67 (33.2)	18 (8.9)	0.250
		Yes	7 (3.5)	33 (16.4)	80 (39.8)	65 (32.3)	16 (8.0)	
	Deep boiled soup	No	59 (29.2)	98 (48.5)	38 (18.8)	5 (2.5)	2 (1.0)	0.759
		Yes	56 (27.9)	104 (51.7)	38 (18.9)	2 (1.0)	1 (0.5)	
	Western soup	No	13 (6.5)	73 (36.3)	81 (40.3)	22 (10.9)	12 (6.0)	0.059
		Yes	18 (9.0)	51 (25.4)	86 (42.8)	37 (18.4)	9 (4.5)	
*Neutral*	Fruits and vegetables	No	44 (21.6)	131 (64.8)	25 (12.6)	2 (1.0)	0 (0.0)	0.452
		Yes	41 (20.2)	121 (60.1)	37 (18.1)	3 (1.6)	0 (0.0)	
	Pork	No	15 (7.5)	95 (46.8)	83 (41.3)	5 (2.5)	4 (2.0)	0.690
		Yes	18 (9.0)	94 (46.5)	86 (42.5)	2 (1.0)	2 (1.0)	
	Pork with fat	No	4 (2.0)	17 (8.4)	70 (34.7)	66 (32.7)	45 (22.3)	0.121
		Yes	4 (2.0)	32 (16.0)	72 (35.5)	63 (31.0)	31 (15.5)	
	Geese/duck	No	5 (2.5)	45 (22.3)	101 (50.0)	36 (17.8)	15 (7.4)	0.209
		Yes	13 (6.5)	55 (27.0)	92 (45.5)	31 (15.5)	11 (5.5)	
	Fresh water fish	No	19 (9.4)	79 (39.1)	80 (39.6)	16 (7.9)	7 (3.5)	0.140
		Yes	10 (5.0)	63 (31.3)	100 (49.3)	20 (10.0)	9 (4.5)	
	Nuts	No	39 (19.5)	84 (41.5)	64 (31.5)	10 (5.0)	5 (2.5)	0.020*
		Yes	26 (12.6)	65 (32.2)	88 (43.7)	18 (9.0)	5 (2.5)	
	Boiling soup	No	37 (18.3)	98 (48.5)	57 (28.2)	6 (3.0)	4 (2.0)	0.233
		Yes	31 (15.5)	90 (44.5)	61 (30.0)	16 (8.0)	4 (2.0)	
*Cold*	Fruits and vegetables	No	38 (19.0)	117 (58.0)	45 (22.5)	1 (0.5)	0 (0.0)	0.323
		Yes	30 (14.9)	11 (56.9)	53 (26.1)	4 (2.1)	0 (0.0)	
	Sea fish	No	35 (17.3)	97 (48.0)	58 (28.7)	10 (5.0)	2 (1.0)	0.963
		Yes	31 (15.5)	102 (50.5)	57 (28.0)	9 (4.5)	3 (1.5)	
	Squid	No	27 (13.6)	59 (29.1)	85 (42.2)	25 (12.6)	5 (2.5)	0.219
		Yes	19 (9.5)	45 (22.1)	96 (47.7)	36 (17.6)	6 (3.0)	
	Products made from bean	No	53 (26.4)	103 (50.7)	39 (19.4)	5 (2.5)	2 (1.0)	*0.019* *
		Yes	39 (19.1)	87 (43.2)	67 (33.2)	8 (4.0)	1 (0.5)	
	Light Chinese tea	No	28 (13.9)	82 (40.6)	64 (31.7)	23 (11.4)	5 (2.5)	0.148
		Yes	33 (16.1)	64 (31.7)	78 (38.7)	26 (13.1)	1 (0.5)	
	Herbal tea	No	4 (2.0)	40 (19.8)	111 (55.0)	39 (19.3)	8 (4.0)	0.582
		Yes	5 (2.5)	36 (17.7)	101 (50.0)	47 (23.2)	13 (6.6)	

The *P* value was <0.05, indicating a statistically significant between-group difference. The results showed a possible association between consumption of *hot* foods and a higher risk of breast cancer

* *P* values were calculated by Chi square tests

**Table 4:** Odds ratio and its corresponding 95% confidence intervals of having breast cancer by eating different food

	Controls N = 202	Cases N = 202	OR	95% CI	*P* value
Shrimp					
Average	84	73	1.00	Reference	
Much preferred	13	44	3.90	1.95–7.79	*<0.001* *
Preferred	53	60	1.30	0.80–2.12	0.285
Not preferred	38	19	0.58	0.31–1.08	0.086
Not preferred much	14	5	0.41	0.14–1.20	0.094
Shellfish					
Average	97	73	1.00	Reference	
Much preferred	9	31	4.58	2.05–10.21	<0.001*
Preferred	37	56	2.01	1.20–3.36	0.007*
Not preferred	48	39	1.08	0.64–1.82	0.773
Not preferred much	11	3	0.36	0.10–1.35	0.116
Soft drinks					
Average	65	72	1.00	Reference	
Much preferred	3	10	3.01	0.79–11.41	0.092
Preferred	10	30	2.71	1.23–5.97	0.011*
Not preferred	92	65	0.64	0.40–1.01	0.056
Not preferred much	31	24	0.70	0.37–1.31	0.264
Milk					
Average	91	90	1.00	Reference	
Much preferred	10	25	2.53	1.15–5.57	0.018*
Preferred	49	37	0.76	0.46–1.28	0.306
Not preferred	35	43	1.24	0.73–2.12	0.425
Not preferred much	17	7	0.42	0.17–1.05	0.058
Popcorn/fries					
Average	79	86	1.00	Reference	
Much preferred	3	15	4.59	1.28–16.46	0.011*
Preferred	34	32	0.87	0.49–1.53	0.618
Not preferred	57	46	0.74	0.45–1.22	0.235
Not preferred much	29	23	0.73	0.39–1.36	0.321
Nuts					
Average	64	88	1.00	Reference	
Much preferred	39	26	0.49	0.27–0.88	0.016*
Preferred	84	65	0.56	0.36–0.89	0.013*
Not preferred	10	18	1.31	0.57–3.02	0.528
Not preferred much	5	5	0.73	0.20–2.62	0.745
Products made from bean					
Average	39	67	1.00	Reference	
Much preferred	53	39	0.43	0.24–0.76	0.003*
Preferred	103	87	0.49	0.30–0.80	0.004*
Not preferred	5	8	0.93	0.29–3.05	1.000
Not preferred much	2	1	0.29	0.026–3.32	0.555

A statistically significant OR (odds ratio) >1 indicates a risk factor; an OR <1 indicates a prevention factor

* indicates a statistically significant between-group diference

### Association between CM dietary patterns or habits and risk of breast cancer

A higher frequency of eating out for breakfast (4–5 times per week, *P* = 0.015; 6–7 times per week, *P* < 0.001) and lunch (4–5 times per week, *P* < 0.001; 6–7 times per week, *P* = 0.006) was positively associated with the risk of breast cancer. In general, participants who dined out for breakfast and lunch (at least four times per week) had at least twice the risk of having breast cancer compared with participants who dined out 0–1 time per week (Table 5). A higher frequency of Guangdong *dim sum*/tea drinking was positively associated with a higher risk of breast cancer. Participants who ate *dim sum* more than three times per week were 2.8 times higher in frequency to be at risk of breast cancer than those who ate it less than once a week (1–2 times per week, *P* = 0.05; >3 times per week, *P* < 0.001) (Table 6).

**Table 5:** Adjusted odds ratio and its corresponding 95% confidence intervals of having breast cancer by dining out patterns (fast food)

	Controls *n* = 202	Cases *n* = 202	Adjusted OR	95% CI	*P* value
Breakfast					
0–1 times per week	113	81	1.00	Reference	
2–3 times per week	39	34	1.22	0.71–2.09	0.479
4–5 times per week	19	30	2.20	1.16–4.18	0.015*
6–7 times per week	31	56	2.52	1.49–4.25	<0.001*
Lunch					
0–1 times per week	68	40	1.00	Reference	
2–3 times per week	56	59	1.70	0.99–2.91	0.053
4–5 times per week	31	44	3.24	1.80–5.80	<0.001*
6–7 times per week	47	59	2.13	1.24–3.69	0.006*

Adjusted for age and body mass index (BMI)

* The *P* value was <0.05, indicating a statistically significant between-group difference

**Table 6:** Adjusted odds ratio and its corresponding 95% confidence intervals of having breast cancer by frequency of Guangdong drinking tea

	Controls *n* = 202	Cases *n* = 202	Adjusted OR	95% CI	*P* value
Guangdong tea (Yum Cha)					
Less than once per week	142	109	1.00	Reference	
1–2 times per week	46	56	1.59	1.00–2.52	0.05*
>3 times per week	12	35	3.80	1.88–7.66	<0.001*

The *P* value was <0.05, indicating a statistically significant between-group difference

* Adjusted for age and body mass index (BMI)

### Consumption of CM supplements, Chinese soup, and risk of breast cancer

The results indicated significant positive associations of the consumption of CM supplements and Chinese soup with a lower risk of breast cancer (Table 7).

**Table 7:** Adjusted odds ratio and its corresponding 95% confidence intervals of having breast cancer by the dietary patterns of Chinese medicine and related supplement and soup intake

	Controls *n* = 202	Cases *n* = 202	Adjusted OR	95% CI	*P* value
Chinese medicine					
Seldom	98	112	1.00	Reference	
Sometimes	61	51	0.73	0.46–1.16	0.183
Often	43	36	0.73	0.44–1.23	0.240
Food supplement					
Seldom	119	140	1.00	Reference	
Sometimes	35	32	0.78	0.45–1.33	0.358
Often	47	26	0.47	0.28–0.81	0.005*
Chinese soup					
Seldom	91	115	1.00	Reference	
Sometimes	83	69	0.66	0.43–1.00	0.051
Often	28	16	0.45	0.23–0.89	0.019*

The *P* value was <0.05, indicating a statistically significant between-group difference

* Adjusted for age and body mass index (BMI)

## Discussion

CM theory offers a way of classifying food based on its properties. When the balance of *Yin* and *Yang* in the body is upset, many diseases can develop, including breast cancer [14, 16]. Although different from modern nutritional classification theories, CM-based food classification could help people to improve their dietary patterns by eating more *neutral* or *cold* pattern foods and reducing consumption of high-risk *hot* foods to prevent breast cancer. Different food characteristics can alter the balance of *Yin* and *Yang* in the body. Numerous experimental studies have demonstrated that some food based on CM classifications may reduce the risk of cancer [8, 16–20]. However, no epidemiological research has reported a relationship between CM-defined dietary patterns and breast cancer morbidity.

In this study, participants with breast cancer shared a preference for a *hot*-patterned diet containing fried foods and meat rather than vegetables. This may have contributed to their breast cancer. The participants with breast cancer also had a relatively higher frequency of dining out, eating fast food, and consuming soft drinks, coffee, milk, and other foods classified as *hot* according to CM theory. In CM, *hot* food is defined as food that is high in energy and low in fiber, such as beef and prawns, or food that is fried or roasted [8, 21]. This is consistent with the etiology and pathogenesis of breast cancer in Western medicine [22]. It was demonstrated that carcinogenic substances found in foods according to Western medicine mostly belong to foods classified as *hot* according to CM, such as barbecued foods, red meat, fatty meat, and prawns [23–27].

Depending on the seasons and individual constitution, people in Hong Kong will choose different foods, herbs, or soups to adjust the functions of the organs and balance the *Yin* and *Yang* of the body [28, 29]. Our findings suggested that frequent consumption of Chinese herbs or Chinese soup is inversely associated with the risk of breast cancer. CM dietary herbs (supplements) or Chinese herbal medicine may play a crucial role in preventing the incidence, recurrence, and metastasis of breast cancer. Many experimental studies have demonstrated that some foods representative of the CM classifications could reduce the risk of cancer [30–32], and that there is a relationship between the use of CM herbs or related Chinese herbal medicine and the reduced incidence of breast cancer [33].

The design and conduct of this study had some limitations. This study did not examine other factors related to breast cancer, such as whether or not participants exercised regularly or whether they used oral contraceptives. In addition, because the control group was recruited from health workshops, and included both healthy and unhealthy women, there may be a health worker effect resulting in selection bias. Because of limited resources, we recruited only 37 people to our pilot study. This sample size was not large enough to conduct a factor analysis to assess the internal consistency of the questionnaire. Because this questionnaire was the first to analyze dietary patterns in terms of *hot*, *neutral*, and *cold* characteristics as defined by CM, there was no other standard questionnaire and results for comparison. The evidence strength in terms of epidemiology is also insufficient.

## Conclusions

Non-breast cancer participants adopted a healthy and balanced (*neutral*) dietary pattern, along with consumption of CM supplements and Guangdong soups.

## Additional files

**Additional file 1.** Classification and characteristics of foods according to TCM theory.

**Additional file 2.** Reference food questionnaire.

**Additional file 3.** Minimum standards of reporting checklist.

**Additional file 4.** Questionnaire-N-dietary.

**Additional file 5.** IRB.

## Abbreviations

CM: Chinese medicine
FFQ: Food Frequency Questionnaires
CSCMHKU: Clinic Center of School of Chinese Medicine, The University of Hong Kong
BMI: body mass index
