# Supplementary material for: Relationship between Chinese medicine dietary patterns and the incidence of breast cancer in Chinese women in Hong Kong: a retrospective cross-sectional survey
Source: Chin Med. 2017 Jun 29;12:17. doi: 10.1186/s13020-017-0138-9 (PMC5492296; doi:10.1186/s13020-017-0138-9)
Supplement: Supplementary file 1 — Additional file 1. Classification and characteristics of foods according to TCM theory. [file 13020_2017_138_MOESM1_ESM.docx]

**Additional File 1.**

**Classification and characteristics of foods according to TCM theory**

|  | ***Cold (cool)*** |  | ***Neutral*** |  | ***Hot (warm)*** |
| --- | --- | --- | --- | --- | --- |
| Meanings | Eat this kind of food for a long time and usually the [corporeity](javascript:void(0)) of hot can be rectified, thus likely to cool the body |  | Between cold and heat, neutral |  | Eat this kind of food for a long time and usually the [corporeity](javascript:void(0)) of cold can be rectified, thus likely to warm the body |
| Function | Clears away heat, purges fire and eliminates toxic materials, which are used for heat-syndrome |  | Provide nutrition to body |  | Expels cold and restores Yang; used for cold syndromes |
| Character | Belongs to *Yin* |  | *Neutral* |  | Belongs to *Yang* |
| Examples | Vegetables*:* Asparagus, sprouts, cauliflower, celery, Chinese cabbage, chrysanthemum flower, cucumber, lotus root, dandelion leaf, seaweed, snow peas, white mushroom. |  | Vegetables*:*  Beans,  bamboo shoots, bitter gourd, mushroom, broccoli, cabbage, carrot, cooked lettuce, corn, daikon radish, potato, soybean sprouts, spinach, swiss chard, tomato. nuts |  | Vegetables*:*  Bell peppers, cooked tomatoes, fennel, |
|  | Fruits:  Banana, blueberry, kiwi, lemon, mulberry, orange, pear, persimmon, watermelon. |  | Fruits:  Apple, avocado, black currant, cherries, fig, prunes |  | Fruits:  Lychee, durian, longan, pomegranate, mango, quince, raspberry |
|  | Grains:  wheat germ, mung bean |  | Grains:  rice barley, buckwheat, millet, wheat |  | Grains*:*  Quinoa, sweet/glutinous rice |
|  | Spices*:*  Salt, peppermint |  | Spices*:*  cilantro leaf, tamarind |  | Spices*:*  Anise, bay leaf, capers, caraway, black pepper, cayenne pepper, chili pepper clove, coriander, cumin, nutmeg |
|  | Misc:  Soy sauce |  | Misc:  Beer, miso soup, sesame oil, yogurt |  | Misc:  Black tea, coffee, milk, wine sheep's milk |
|  | Meat:  Most sea fish, tortoise |  | Meat*:*  Duck egg, duck, frog, pork, rabbit |  | Meat*:*  Beef, butter, capers, chicken, eel, ham, lobster, mutton, lamb, shrimp, turkey. |

According to CM theory and the famous work from Lisizhen《Compendium of Material medica》,《Pharmacopoeia of the People’s Republic of China》 there are three (detail is five) types of nature food classified as “*cold*（*cool*）” “*neutral*” and “*ho*t（*warm*）” . They are not dependent on the temperature of the food, but the nature or [property](javascript:void(0)) or/and effect of the food itself which shows different cold and hot properties.
