# Supplementary material for: Relationship between Chinese medicine dietary patterns and the incidence of breast cancer in Chinese women in Hong Kong: a retrospective cross-sectional survey
Source: Chin Med. 2017 Jun 29;12:17. doi: 10.1186/s13020-017-0138-9 (PMC5492296; doi:10.1186/s13020-017-0138-9)
Supplement: Supplementary file 2 — Additional file 2. Reference food questionnaire. [file 13020_2017_138_MOESM2_ESM.pdf]

Food Questionnaire

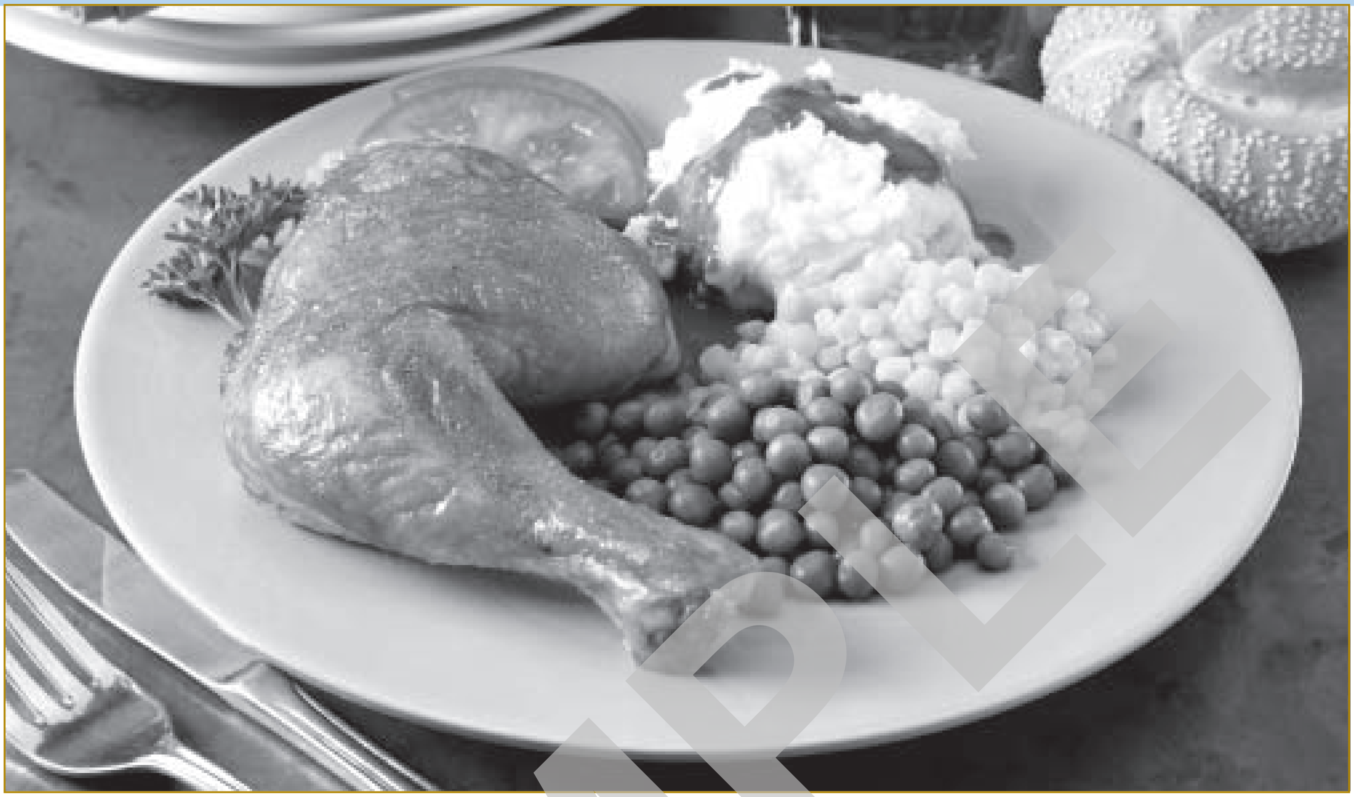

SAMPLE

This form asks about your usual food intake during ______________________________ .

Please use **pencil**.

Answer by filling in the correct oval.

● Yes ○ No

Do not make any other marks on the form. Please use a separate piece of paper to make comments.

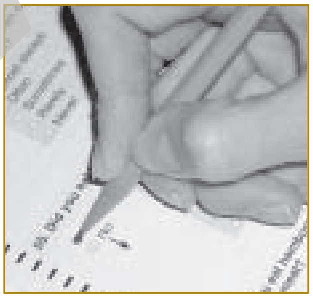

SEX
○ Male
○ Female

TODAY'S DATE							
MO		DAY		YEAR			
0	0	0	0	0	0	0	0
1	1	1	1	1	1	1	1
	2	2	2	2	2	2	2
	3	3	3	3	3	3	3
	4		4	4	4	4	4
	5		5	5	5	5	5
	6		6	6	6	6	6
	7		7	7	7	7	7
	8		8	8	8	8	8
	9		9	9	9	9	9

IDENTIFICATION NUMBER								
0	0	0	0	0	0	0	0	0
1	1	1	1	1	1	1	1	1
2	2	2	2	2	2	2	2	2
3	3	3	3	3	3	3	3	3
4	4	4	4	4	4	4	4	4
5	5	5	5	5	5	5	5	5
6	6	6	6	6	6	6	6	6
7	7	7	7	7	7	7	7	7
8	8	8	8	8	8	8	8	8
9	9	9	9	9	9	9	9	9

GNA

PLEASE DO NOT WRITE IN THIS AREA

Part I: Usual Food Choices

These questions are about the types of foods you ate during ________________________________.

1. Did you eat chicken or turkey?

- Yes → **When you ate chicken or turkey, how often did you eat the skin?**
  - Almost always
  - Often
  - Sometimes
  - Rarely
  - Never
- No ↓

2. Did you eat beef, pork, ham or lamb?

- Yes → **When you ate beef, pork, ham or lamb, how often did you eat the fat?**
  - Almost always
  - Often
  - Sometimes
  - Rarely
  - Never
- No ↓

3. Did you eat hamburger or other ground meat?

- Yes → **When you ate hamburger or other ground meat, was it usually...** *Mark one or two.*
  - Regular
  - Lean
  - Extra lean
  - Ground chicken or turkey
  - Don't know
- No ↓

4. Did you drink orange, grapefruit or other fruit juices?

- Yes → **Were any of these vitamins or minerals added (specially fortified) to the juices you drank?** *Mark all that apply.*
  - Extra Vitamin C
  - Vitamin E
  - Calcium
  - None
  - Don't know
- No ↓

5. Did you eat cold cereals?

- Yes → **When you ate cold cereal, what type did you usually eat?** *Mark one or two.*
  - Highly fortified cereals (100% of Daily Values) such as Total®, Smart Start® and Product 19®
  - High fiber or bran cereals such as Raisin Bran® and All Bran®
  - Sweetened cereals such as Frosted Flakes® and Froot Loops®
  - All other cereals such as Cheerios®, Corn Flakes® and granola
- No ↓

6. Did you put milk (all types), cream or creamer on cereal?

- Yes → **When you put milk, cream or creamer on cereal, what type did you usually use?** *Mark one or two.*
  - Cream or half and half
  - Whole milk
  - 2% milk
  - 1% milk or buttermilk
  - Nonfat or skim milk
  - Soy milk
  - Non-dairy creamer
  - Don't know
- No ↓

SAMPLE

7. Did you put milk (all types), cream or creamer in coffee or tea?

- Yes → **When you put milk, cream or creamer in coffee or tea, what type did you usually use?** *Mark one or two.*
  - Cream or half and half
  - Whole milk
  - 2% milk
  - 1% milk or buttermilk
  - Nonfat or skim milk
  - Soy milk
  - Non-dairy creamer
  - Don't know
- No ↓

8. Did you drink milk (all types)? *Also include beverages made with milk, such as lattes, cappuccinos, mochas or hot chocolate.*

- Yes → **When you drank milk or beverages made with milk, was it usually...** *Mark one or two.*
  - Whole milk
  - 2% milk
  - 1% milk or buttermilk
  - Nonfat or skim milk
  - Soy milk
  - Don't know
- No ↓

9. Did you use salad dressing?

- Yes → **When you used salad dressing, what type did you usually use?** *Mark one or two.*
  - Regular, including oil and vinegar
  - Low or reduced fat
  - Fat free or nonfat
- No ↓

10. Did you use mayonnaise?

- Yes → **When you used mayonnaise, what type did you usually use?** *Mark one or two.*
  - Regular
  - Low or reduced fat
  - Fat free or nonfat
- No ↓

11. Did you eat cookies or cakes?

- Yes → **When you ate cookies or cakes, how often were they fig bars, angel food cakes, or other types of low or nonfat cookies or cakes?**
  - Almost always
  - Often
  - Sometimes
  - Rarely
  - Never
- No ↓

12. In your household, what kinds of fat were usually used when cooking, for example to flavor vegetables or fry meat? *Mark up to four.*

- Butter
- Butter blended with oil or margarine
- Stick margarine
- Regular tub margarine
- Diet or light margarine (tub or liquid)
- Olive oil
- Canola oil
- Other oils such as corn, soybean, peanut and safflower
- Lard, bacon fat or meat drippings
- Didn't use fat or used non-stick spray (Pam®)

13. What kinds of fat did you use at the table, for example on breads, vegetables or potatoes? *Mark up to four.*

- Butter
- Butter blended with oil or margarine
- Stick margarine
- Regular tub margarine
- Diet or light margarine (tub or liquid)
- Olive oil
- Sour cream
- Didn't use fat

SAMPLE

PLEASE DO NOT WRITE IN THIS AREA

27062- 2-3

Part II: Usual Food Use

These questions are about foods you ate during ______________________________.

14. Mark the column to show how often, on average, you ate the following foods. Mark your usual serving size as small, medium or large.

- A small serving is about one-half (1/2) the medium serving size or less.
- A large serving is about one-and-a-half (1 1/2) times the medium serving size or more.

EXAMPLE: This person ate spaghetti with meat sauce every Saturday. They usually ate about 1 1/2 cups.

	HOW OFTEN DID YOU EAT THESE FOODS?									→	AMOUNT?		
	**NEVER or less than once per month**	**1** per month	**2-3** per month	**1** per week	**2** per week	**3-4** per week	**5-6** per week	**1** per day	**2+** per day	**Medium serving size**	**S**	**M**	**L**
Spaghetti, lasagna, and other pasta with tomato and meat sauce	○	○	○	●	○	○	○	○	○	1 cup	○	○	●

CEREALS, BREADS, SNACKS

	HOW OFTEN DID YOU EAT THESE FOODS?									→	AMOUNT?		
	**NEVER or less than once per month**	**1** per month	**2-3** per month	**1** per week	**2** per week	**3-4** per week	**5-6** per week	**1** per day	**2+** per day	**Medium serving size**	**S**	**M**	**L**
Cold cereals	○	○	○	○	○	○	○	○	○	1 cup	○	○	○
Cooked cereals and grits	○	○	○	○	○	○	○	○	○	1cup	○	○	○
Milk on cereals	○	○	○	○	○	○	○	○	○	1/2 cup	○	○	○
Pancakes, French toast and waffles	○	○	○	○	○	○	○	○	○	2 pieces	○	○	○
Muffins, scones, croissants and biscuits	○	○	○	○	○	○	○	○	○	1 medium	○	○	○
White breads, including bagels, rolls and English muffins	○	○	○	○	○	○	○	○	○	2 slices or 1 medium	○	○	○
Whole grain breads and rolls	○	○	○	○	○	○	○	○	○	2 slices or 1 medium	○	○	○
Plain tortillas as a side dish (include flour and corn)	○	○	○	○	○	○	○	○	○	2 small or 1 medium	○	○	○
Cornbread and corn muffins	○	○	○	○	○	○	○	○	○	2 slices or 1 medium	○	○	○
Butter or margarine on breads, cereals, pancakes, etc.	○	○	○	○	○	○	○	○	○	2 pats or 2 teaspoons	○	○	○
Jam, jelly, honey, syrup and sugar (including in coffee, tea and cereal)	○	○	○	○	○	○	○	○	○	2 Tbsp.	○	○	○
Granola bars and cereal bars such as Nutri-Grain Bars®	○	○	○	○	○	○	○	○	○	1 bar	○	○	○
Sports or meal replacement bars such as Power Bars® and Clif Bars®	○	○	○	○	○	○	○	○	○	1 bar	○	○	○

SAMPLE

CEREALS, BREADS, SNACKS (continued)

	HOW OFTEN DID YOU EAT THESE FOODS?									→	AMOUNT?		
	NEVER or less than once per month	1 per month	2-3 per month	1 per week	2 per week	3-4 per week	5-6 per week	1 per day	2+ per day	Medium serving size	S	M	L
Low or nonfat potato chips, tortilla chips, corn chips and pretzels	○	○	○	○	○	○	○	○	○	2 handfuls or 1 sm. bag	○	○	○
Regular potato chips, tortilla chips, corn chips and puffs	○	○	○	○	○	○	○	○	○	2 handfuls or 1 sm. bag	○	○	○
Plain popcorn (no butter) or lowfat microwave popcorn	○	○	○	○	○	○	○	○	○	4 handfuls	○	○	○
Buttered or regular microwave popcorn	○	○	○	○	○	○	○	○	○	4 handfuls	○	○	○
Low or nonfat crackers such as saltines	○	○	○	○	○	○	○	○	○	6 medium	○	○	○
Whole grain crackers such as Triscuits® and rye crispbread	○	○	○	○	○	○	○	○	○	6 medium	○	○	○
Regular crackers such as Ritz® and club crackers	○	○	○	○	○	○	○	○	○	6 medium	○	○	○
Peanut butter, peanuts and other nuts and seeds	○	○	○	○	○	○	○	○	○	2 Tbsp. (spreads) or 1/4 cup (nuts)	○	○	○

MEAT, FISH, EGGS

	HOW OFTEN DID YOU EAT THESE FOODS?									→	AMOUNT?		
	NEVER or less than once per month	1 per month	2-3 per month	1 per week	2 per week	3-4 per week	5-6 per week	1 per day	2+ per day	Medium serving size	S	M	L
Eggs (egg substitute, mark "NEVER")	○	○	○	○	○	○	○	○	○	2 eggs	○	○	○
Bacon and breakfast sausage	○	○	○	○	○	○	○	○	○	3 strips or 2 links	○	○	○
Low or reduced fat hot dogs and sausage	○	○	○	○	○	○	○	○	○	1 hot dog or 2 ounces	○	○	○
Regular hot dogs and sausage such as bratwurst and chorizo	○	○	○	○	○	○	○	○	○	1 hot dog or 2 ounces	○	○	○
Lunch meats such as ham, turkey and lowfat bologna	○	○	○	○	○	○	○	○	○	2 slices	○	○	○
All other lunch meat such as bologna, salami and Spam®	○	○	○	○	○	○	○	○	○	2 slices	○	○	○
Canned tuna, tuna salad and tuna casserole	○	○	○	○	○	○	○	○	○	1/2 can tuna or 1 cup casserole	○	○	○
Beef, pork, ham and lamb	○	○	○	○	○	○	○	○	○	4 ounces	○	○	○
Ground meat, including hamburgers and meatloaf	○	○	○	○	○	○	○	○	○	1 medium patty or 3 ounces	○	○	○
Liver, chicken liver and organ meats	○	○	○	○	○	○	○	○	○	4 ounces	○	○	○
Fried chicken, including nuggets and tenders	○	○	○	○	○	○	○	○	○	1 large piece or 6 nuggets	○	○	○

SAMPLE

PLEASE DO NOT WRITE IN THIS AREA

27062- 3-3

MEAT, FISH, EGGS (continued)

	HOW OFTEN DID YOU EAT THESE FOODS?									→	AMOUNT?		
	NEVER or less than once per month	1 per month	2-3 per month	1 per week	2 per week	3-4 per week	5-6 per week	1 per day	2+ per day	Medium serving size	S	M	L
Chicken and turkey (roasted, stewed, grilled or broiled)										1 large or 2 small pieces			
Fried fish, fish sandwich and fried shellfish (shrimp and oysters)										3 ounces or 1 sandwich			
Shellfish, not fried (shrimp, lobster, crab and oysters)										3 ounces or ½ cup			
White fish (broiled or baked) such as sole, halibut, snapper and cod										4 ounces			
Dark fish (broiled or baked) such as salmon, mackerel and bluefish										4 ounces			

SPAGHETTI, MIXED DISHES, SOUPS

	HOW OFTEN DID YOU EAT THESE FOODS?									→	AMOUNT?		
	NEVER or less than once per month	1 per month	2-3 per month	1 per week	2 per week	3-4 per week	5-6 per week	1 per day	2+ per day	Medium serving size	S	M	L
Stew, pot pie, curries and casseroles with meat or chicken										1 cup			
Chili with meat and beans										1 cup			
Spaghetti, lasagna and other pasta with tomato and meat sauce										1 cup			
Spaghetti and other pasta with tomato sauce (no meat)										1 cup			
Pasta with oil, cheese, or cream sauce, including macaroni and cheese										1 cup			
Asian-style (stir-fried) noodles and rice such as chow mein, fried rice and Pad Thai										1 cup			
Pizza										2 slices			
Tofu, tempeh and products such as tofu hot dogs, soy burgers and tofu cheese										3 ounces, 1 hot dog or 1 burger			
Burritos, tacos, tostadas and quesadillas										1 medium			
Enchiladas and tamales										1 medium			
Vegetable, minestrone and tomato soup										1 cup			
Cream soups such as chowders, potato and cheese										1 cup			

SAMPLE

SPAGHETTI, MIXED DISHES, SOUPS (continued)

	HOW OFTEN DID YOU EAT THESE FOODS?									→	AMOUNT?		
	NEVER or less than once per month	1 per month	2-3 per month	1 per week	2 per week	3-4 per week	5-6 per week	1 per day	2+ per day	Medium serving size	S	M	L
Bean soups such as pea, lentil and black bean	○	○	○	○	○	○	○	○	○	1 cup	○	○	○
Miso soup	○	○	○	○	○	○	○	○	○	1 cup	○	○	○
Ramen noodle soup	○	○	○	○	○	○	○	○	○	1 cup	○	○	○
Other soups such as chicken noodle	○	○	○	○	○	○	○	○	○	1 cup	○	○	○

DAIRY PRODUCTS

	HOW OFTEN DID YOU EAT THESE FOODS?									→	AMOUNT?		
	NEVER or less than once per month	1 per month	2-3 per month	1 per week	2 per week	3-4 per week	5-6 per week	1 per day	2+ per day	Medium serving size	S	M	L
Cottage cheese and ricotta cheese	○	○	○	○	○	○	○	○	○	1/2 cup	○	○	○
Low or reduced fat cheese, including cheese used in cooking	○	○	○	○	○	○	○	○	○	1 slice or 1/4 cup shredded	○	○	○
All other cheese (American, cheddar or cream), including cheese used in cooking	○	○	○	○	○	○	○	○	○	1 slice, 1/4 cup shredded or 2 Tbsp. cream	○	○	○
Yogurt, all types except frozen	○	○	○	○	○	○	○	○	○	6 ounces	○	○	○

VEGETABLES and GRAINS

	HOW OFTEN DID YOU EAT THESE FOODS?									→	AMOUNT?		
	NEVER or less than once per month	1 per month	2-3 per month	1 per week	2 per week	3-4 per week	5-6 per week	1 per day	2+ per day	Medium serving size	S	M	L
Mark all vegetables you ate, including in salads, mixed dishes, sandwiches and stir-fries.													
Green salad (lettuce or spinach)	○	○	○	○	○	○	○	○	○	1 cup	○	○	○
Salad dressing (all types)	○	○	○	○	○	○	○	○	○	2 Tbsp.	○	○	○
Fresh tomatoes	○	○	○	○	○	○	○	○	○	1 medium or 4 slices	○	○	○
Carrots	○	○	○	○	○	○	○	○	○	1/2 cup	○	○	○
Green peppers and green chilies	○	○	○	○	○	○	○	○	○	1/4 cup	○	○	○
Red peppers and red chilies	○	○	○	○	○	○	○	○	○	1/4 cup	○	○	○

SAMPLE

VEGETABLES and GRAINS (continued)

	HOW OFTEN DID YOU EAT THESE FOODS?								→	AMOUNT?		
	NEVER or less than once per month	1 per month	2-3 per month	1 per week	2 per week	3-4 per week	5-6 per week	1 per day	2+ per day	Medium serving size	S	M	L
Mark all vegetables you ate, including in salads, mixed dishes, sandwiches and stir-fries.													
Broccoli										1/2 cup			
Cauliflower, cabbage and Brussels sprouts										1/2 cup			
Green or string beans										1/2 cup			
Green peas										1/2 cup			
Corn and hominy										1/2 cup			
Summer squash and zucchini										1/2 cup			
Winter squash such as acorn, butternut and pumpkin										1/2 cup			
Yams and sweet potatoes										1 medium			
Cooked greens such as spinach, mustard greens and collards										1/2 cup			
Onions and leeks										1/4 cup			
Fresh garlic, including in cooking										1 clove			
Avocado and guacamole										1/4 medium or 1/4 cup			
French fries, fried potatoes and hash browns										3/4 cup			
Potatoes (boiled, baked or mashed)										1 medium or 3/4 cup			
Refried beans										1/2 cup			
All other beans (baked, lima or chili without meat)										1/2 cup			
Coleslaw										1/2 cup			
Potato, macaroni and pasta salads made with mayonnaise or oil										1/2 cup			
Brown rice, whole wheat pasta and other whole grains (as a side dish)										1 cup			
White rice, noodles and other grains (as a side dish)										1 cup			
Butter, margarine, sour cream and other fat added to vegetables, potatoes and rice										1 pat or 1 teaspoon			

SAMPLE

PLEASE DO NOT WRITE IN THIS AREA

SAUCES and CONDIMENTS

	HOW OFTEN DID YOU EAT THESE FOODS?									→	AMOUNT?		
	NEVER or less than once per month	1 per month	2-3 per month	1 per week	2 per week	3-4 per week	5-6 per week	1 per day	2+ per day	Medium serving size	S	M	L
Cheese sauce and cream sauce										$\frac{1}{4}$ cup			
Meat gravies										$\frac{1}{4}$ cup			
Ketchup										2 Tbsp.			
Salsa (as dip or on foods)										$\frac{1}{4}$ cup			
Mayonnaise and mayonnaise-type spreads										2 Tbsp.			

FRUITS

	HOW OFTEN DID YOU EAT THESE FOODS?									→	AMOUNT?		
	NEVER or less than once per month	1 per month	2-3 per month	1 per week	2 per week	3-4 per week	5-6 per week	1 per day	2+ per day	Medium serving size	S	M	L
Apples, applesauce and pears										1 medium or $\frac{1}{2}$ cup			
Bananas										1 medium			
Peaches, nectarines and plums										1 medium or $\frac{1}{2}$ cup			
Apricots (fresh, canned or dried)										2 medium or 4 halves			
Dried fruit (other than apricots) such as raisins and prunes										$\frac{1}{4}$ cup			
Oranges, grapefruit and tangerines (not juice)										1 orange or $\frac{1}{2}$ grapefruit			
Berries such as strawberries and blueberries										$\frac{1}{2}$ cup			
Cantaloupe, orange melon and mango										$\frac{1}{4}$ melon or $\frac{1}{2}$ mango			
Watermelon and red melon										1 medium slice			
Any other fruit such as grapes, fruit cocktail, pineapple and cherries										$\frac{1}{2}$ cup			

SAMPLE

SWEETS

	HOW OFTEN DID YOU EAT THESE FOODS?									→	AMOUNT?		
	**NEVER or less than once per month**	**1** per month	**2-3** per month	**1** per week	**2** per week	**3-4** per week	**5-6** per week	**1** per day	**2+** per day	**Medium serving size**	**S**	**M**	**L**
Low or nonfat frozen desserts such as lowfat ice cream, frozen yogurt and sherbet										1 scoop			
Ice cream and milkshakes										1 scoop or 1 shake			
Pudding, custard and flan										3/4 cup			
Doughnuts, pies and pastries										1 medium piece or slice			
Cookies and cakes										2 med. cookies or 1 piece of cake			
Chocolate, candy bars and toffee										1 regular bar or 2 pieces			
Other candy such as Lifesavers®, licorice and jelly beans										4 pieces or 12 jellybeans			

PLEASE ANSWER THESE THREE IMPORTANT QUESTIONS!

Note that the frequency headings are different.

	**NEVER or less than once per week**	**1-2** per week	**3-4** per week	**5-6** per week	**1** per day	**2** per day	**3** per day	**4** per day	**5+** per day
How often did you eat foods that were cooked in fat (pan-fried, sautéed, or deep-fried)? *Count all fat such as margarine, butter, oil or lard.*									
How often did you eat a serving of vegetables? *Do not count potatoes, salad or beans.*									
How often did you eat a serving of fruit? *Do not count juices.*									

SAMPLE

PLEASE DO NOT WRITE IN THIS AREA

BEVERAGES and ALCOHOL

HOW OFTEN DID YOU DRINK THESE BEVERAGES? → AMOUNT?

Note that the frequency headings are different.

	NEVER or less than once per month	1-3 per month	1 per week	2-4 per week	5-6 per week	1 per day	2-3 per day	4-5 per day	6+ per day	Medium serving size	S	M	L
Milk (all types) as a beverage										1 cup			
Latte, cappuccino, mocha or hot chocolate										1 cup			
Coffee (not lattes or mochas)										1 cup			
Tea, unsweetened or diet										1 cup			
Tea, presweetened, bottled or instant										1 cup			
Milk, cream or creamer added to tea and coffee										1 Tbsp.			
Tomato juice, V-8® and other vegetable juices										1 cup			
Orange juice and grapefruit juice										1 cup			
Other 100% fruit juice such as apple, grape and cranberry										1 cup			
Fruit drinks fortified with Vitamin C such as Hi-C®, and Kool-Aid®										1 cup			
Meal replacement drinks and shakes such as Slim-Fast® and Ensure®										1 cup			
Diet soft drinks (include energy drinks)										12 ounces or 1 can			
Regular soft drinks (include energy drinks)										12 ounces or 1 can			
Water (tap, bottled or sparkling)										1 cup			
Beer (all types)										12 ounce can or bottle			
Red wine										1 medium glass (6 oz)			
White or rosé wine										1 medium glass (6 oz)			
Liquor and mixed drinks										1 shot (1½oz) or 1 mixed drink			

SAMPLE

THANK YOU!

Please take a moment to fill in any questions you may have skipped.

SAMPLE

PLEASE DO NOT WRITE IN THIS AREA
