# Supplementary material for: Relationship between Chinese medicine dietary patterns and the incidence of breast cancer in Chinese women in Hong Kong: a retrospective cross-sectional survey
Source: Chin Med. 2017 Jun 29;12:17. doi: 10.1186/s13020-017-0138-9 (PMC5492296; doi:10.1186/s13020-017-0138-9)
Supplement: Supplementary file 4 — Additional file 4. Questionnaire-N-dietary. [file 13020_2017_138_MOESM4_ESM.pdf]

Questionnaire

The University of Hong Kong

N

Dietary Questionnaire(飲食問卷)

All questions contained in this questionnaire are strictly confidential. Data collected will be used for the study undertaken by the School of Chinese Medicine, University of Hong Kong.

This questionnaire contains (問卷內容包括): Part A: General information(一般個人情況)
Part B: Gynaecologic information (婦科相關資料)
Part C: Dietary habits(飲食習慣)

個人資料(此部分內容絕對保密)

Part A 個人資料(此部分內容絕對保密)

1．姓名 _______________編號(內部用):_B______就診診日期(內部用) ___________

2．出生日期(YYYY/MM/DD/) ______________年齡 _____

3．種族_____

4．電話_______________

5．病前體重 ______(kg), 目前體重_____(kg), 身高_____(cm) 體重指數______(Kg/ cm^2)

6． 職業, A☐全職(何種職業__________) B☐兼職 (何種______) C ☐家庭主婦/無工作 D ☐ 其他

7．教育程度：A ☐小學或以下 B ☐中學 C ☐大專或以上 D ☐ 其他

8．宗教：無☐；是☐: A☐基督/天主 B☐佛/道教 C☐拜神/拜祖 D ☐其他

9. 家庭收入 A☐：好(人均 HK$ 3 萬以上) B☐ 中(人均 HK$ 1.5 萬左右) C☐ 一般((人均 HK$ 1 萬左右) D☐ 下(人均 HK$ 6 仟 以下)

Part B Gynaecologic information (婦科相關資料)

Questionnaire

1. .目前的月經狀況____

2. 婚姻狀況： A □單身 B□已婚/同居 C□居霜/分居/離異 (發病前多少___年)

3. 婚姻生活：A□愉快 B□ 一般 C□ 不愉快 D□ 曾經不愉快

4. 性伴侶（異性朋友）：A□愉快 B□ 一般 C□ 不愉快 D□ 曾經不愉快

5. 生育情況：A 已生育小孩，□胎； **B** 初產年齡____ C□未生育原因：無生育能力/其他因素

6. 哺乳情況： □小於 1 月, □大於 1 月; □無

7. 避孕方法：A.□安全套 B.□ 結紮 C.□ 避孕丸 D.□ 其他

8. 腫瘤的遺傳趨象(與問卷者親屬關係/何種腫瘤)

   A. 父系（腫瘤類別） ___________ ___________ ___________ ___________
   B. 母系（腫瘤類別） ___________ ___________ ___________ ___________
   C. 兄弟姐妹（腫瘤類別）___________ ___________ ___________ ___________

Questionnaire

Part C: Dietary habits(飲食習慣)

A. Dietary Habit 飲食習慣

1.你曾否約見過營養師? □有 □沒有.

2.飲食偏嗜(只可選一個)：A□肉類 B□蔬菓 C □無特殊 D□特殊（請註明____________）

3.偏嗜, ---------目前有無改變? A□有 B□稍有 C□沒有

4.你的食慾欲如何：A□很好 B□一般 C□差

5.你有否服用營養輔助品? A□ 有（例如________） B □一點（例如________） C□沒有

6.你有否經常服用中藥? A□有 B □稍有 C□沒有

7. 如有請告訴哪一類藥(例如當歸，川芎) ___________________ ？

8.是否經常煲飲藥膳湯？A□有 B □稍有 C□沒有

9.如有,請告訴用哪一類中藥煲湯？__________________

10. 請列出每次中藥用量?大約____克 ，服用頻率_（_______天 _____ 次）

11. 常用的食油

A□動物油 **B**□粟米/花生油/菜籽油/ C□ 橄欖油 D□葵花籽油 E□其他（請註明_______）

12 · 做每款菜用多少油? A□ 走油 B□1 茶匙或以下 C□ 2 茶匙 D□ 3 茶匙 E □> 3 茶匙

13.你喜歡吃辛辣的食物嗎?

A□ 非常喜歡 B□喜歡 C □一般 D□不喜歡 E□非常不喜歡

14. 平均每星期有多少天出外用餐,

早餐	□ 0	□ 1	□ 2	□ 3	□ 4	□ 5	□ 6	□ 7
午餐	□ 0	□ 1	□ 2	□ 3	□ 4	□ 5	□ 6	□ 7
晚餐	□ 0	□ 1	□ 2	□ 3	□ 4	□ 5	□ 6	□ 7

15. 到茶樓飲茶的平均次數/ 每周 ？

A□0-1 次 B□ 1 次 C□ 2 次 D □3 次 E □ 4 次以上

Questionnaire

B 請形容你的飲食習慣

此部分如有疑問(可與諮詢員聯繫) 請在適合的空格劃上√ 號					
	飲食習慣(经常食用的频率)				
	非常喜歡	喜歡	一般	不喜歡	非常不喜歡
**水果**					
龍眼					
榴蓮					
芒果					
荔枝					
蘋果					
奇異果					
香蕉					
無花果					
木瓜					
棗子					
橙					
山竹					
梨					
西瓜					
枇杷					
柿					
**調料**					
芫荽					
胡椒					
大蒜					
韭菜					
辣椒、 姜					
咖哩					
**瓜菜**					
芥菜					
蕃茄					
南瓜					
薯仔					
芹菜					
白菜					

Questionnaire

飲食習慣(经常食用的频率)					
	非常喜歡	喜歡	一般	不喜歡	非常不喜歡
菇類					
紅蘿蔔					
苦瓜					
西蘭花					
蘿蔔					
蓮藕					
海帶，海藻					
馬蹄					
慈姑					
芥藍					
蘆筍					
其他					
<u>**肉類**</u>					
牛肉類					
羊肉					
雞（請選最喜歡部位<br>1 胸□　2 腿□<br>3 雞翅□）					
豬肉(瘦肉、排骨)					
豬手、肥肉等					
燒鵝/鴨/鴿					
其他					
<u>**魚及海鮮類**</u>					
三文，吞拿					
淡水魚					
鹹水魚					
大蝦					
蟹					
魷魚/墨魚					
貝殼類					
其他類					
**飲食習慣飲食習慣(经常食用的频率)**					

Questionnaire

	非常喜歡	喜歡	一般	不喜歡	非常不喜歡
豆製品及果仁類					
豆製品（豆腐、豆漿、豆芽、）					
多種果仁					
飲品					
發酵茶(普耳，烏龍、鐵觀音)					
綠茶(茶如龍井、香片)					
咖啡，					
奶茶					
汽水					
牛奶類					
高濃度酒的中國白酒、白蘭地					
低酒：如啤酒，葡萄酒					
點心及小吃					
爆穀，薯條					
乳酪					
巧克力					
糖果類					
其他					
雪糕					
熱飲					
凍品					
燻、炸、燒烤物					
湯水					
滾湯類，例如：蔬菜湯；蕃茄湯					
老火湯					
藥膳湯					
西湯（忌廉湯，羅宋湯）					
其他:					
