# Supplementary material for: Relationship between Chinese medicine dietary patterns and the incidence of breast cancer in Chinese women in Hong Kong: a retrospective cross-sectional survey
Source: Chin Med. 2017 Jun 29;12:17. doi: 10.1186/s13020-017-0138-9 (PMC5492296; doi:10.1186/s13020-017-0138-9)
Supplement: Supplementary file 5 — Additional file 5. IRB. [file 13020_2017_138_MOESM5_ESM.pdf]

香港大學
University of Hong Kong
醫院管理局
HOSPITAL AUTHORITY
香港大學及醫管局港島西醫院聯網研究倫理委員會

Institutional Review Board of the University of Hong Kong/ Hospital Authority Hong Kong West Cluster (HKU/HA HKW IRB)

19 AUG 2005

Address: Rm 901, Administration Block, QMH Tel 2855 3351 2855 3923 2855 4086 Fax 2855 4735

Copied to Dr. Chan J.P

Prof. Yao Tong
Dept. of School of Chinese Medicine
University of Hong Kong
18-Aug-05

Dear Prof. Tong,

IRB Reference Number: **UW 05-211 T/874**

The HKU/HA HKW IRB is authorized by the Cluster Chief Executive and the University of Hong Kong, Faculty of Medicine to review and monitor clinical research. It serves to ensure that research complies with the Declaration of Helsinki and acts in accordance to ICH GCP guidelines, local regulations and HA [and the University] policy. It has the authority to approve, require modifications in (to secure approval), or disapprove research. This Committee has power to terminate/suspend a research at any time if there is evidence to indicate that the above principles and requirements have been violated.

Professor C L Lai, Chairman of the HKU/HA HKW IRB has approved your submission on the date shown below by an expedited process. You are required to adhere to the conditions listed.

Date of expedited review (Date/Month/Year) : 12-08-2005

Protocol title : Dietary pattern and changes in life style associated with onset of breast cancer in HK - An epidemiological and health care promotion project

Study site(s) : As stated in application form

Document(s) approved :
01. Clinical research ethics review application form
02. Health care and promotion fund committee (Form P - for non-research health promotion project) and proposed project
03. Information questionnaire and consent form - Chinese

Document(s) reviewed : 04. Short CV of principal investigator

Conditions :
1. Do not deviate from, or make changes to the study protocol without prior written IRB approval, except when it is necessary to eliminate immediate hazards to research subjects or when the change involves only logistical or administrative issues.
2. Report the following to HKU/HA HKW IRB: (i) study protocol or consent document change (use 'HKU/HA HKW IRB RE001F7'), (ii) serious adverse event (use 'HKU/HA HKW IRB RE001F8'). (iii) study progress (use 'HKU/HA HKW IRB RE001F9a')* (iv) new information that may be relevant to a subject's willingness to continue participation in the study.
3. Report first study progress to HKU/HA HKW IRB at a 12-monthly interval until study closure.

(Mr. Chris Yip)
Secretary
HKU/HA HKW IRB

香港大學
University of Hong Kong

醫院管理局
HOSPITAL AUTHORITY

香港大學及醫管局港島西醫院聯網研究倫理委員會
Institutional Review Board of the University of Hong Kong/ Hospital Authority Hong Kong West Cluster (HKU/HA HKW IRB)

Address: Rm 901, Administration Block, QMH Tel 2855 3351 2855 4086 2855 3923 Fax 2855 4735

Prof. Yao Tong
Dept. of School of Chinese Medicine
University of Hong Kong

07-12-2007

Dear Prof. Tong,

IRB Reference Number: **UW 05-211 T/874**

The HKU/HA HKW IRB is authorized by a joint agreement of the University of Hong Kong and Hospital Authority Hong Kong West Cluster to review and monitor clinical research. It serves to ensure that research complies with the Declaration of Helsinki and acts in accordance to ICH GCP guidelines, local regulations and Hospital Authority and the University policies.

I write to inform that your research submission has been approved by an expedited process with details shown below.

**Protocol title**	:	Dietary pattern and changes in life style associated with onset of breast cancer in HK - An epidemiological and health care promotion project
**Date of expedited review**	:	07-12-2007 (Date/Month/Year)
**IRB reviewer**	:	Professor CL Lai, Chairman of the HKU/HA HKW IRB
**Document(s) reviewed**	:	01. Research progress report form dated 2007-11-21
**Note**	:	Please be reminded that principal investigator is responsible for submitting an application for continuing review at a 12-monthly interval until study closure.

Yours sincerely,

W. H. Lee
HKU/HA HKW IRB Secretary
